# The WEAR-BOT checklist: A risk of bias tool for evaluating validity and reliability research in wearable technology

**DOI:** 10.1371/journal.pone.0338014

**Published:** 2026-02-09

**Authors:** Bryson Carrier, Jennifer A. Bunn, Chris Eschbach, Joel D. Reece, Gregory J. Welk, Brett A. Dolezal, James W. Navalta

**Affiliations:** 1 Department of Kinesiology, University of Nevada, Las Vegas, Las Vegas Nevada, United States of America; 2 Department of Natural Sciences, Oregon Tech, Klamath Falls, Oregon, United States of America,; 3 Sam Houston State University, College of Health Sciences, Huntsville, Texas, United States of America; 4 12^th^ State Nutrition, Raleigh, North Carolina, United States of America; 5 Brigham Young University Hawaii, Faculty of Sciences, Laie, Hawaii, United States of America; 6 Department of Kinesiology, Iowa State University, Ames, Iowa, United States of America; 7 David Geffen School of Medicine, University of California, Los Angeles, California, United States of America; Portugal Football School, Portuguese Football Federation, PORTUGAL

## Abstract

This paper proposes an innovative tool designed to standardize the evaluation of validity and reliability studies in the rapidly evolving field of wearable technology. We introduce the WEArable Technology Risk of Bias and Objectivity Tool (WEAR-BOT), a tool that addresses the need for a comprehensive and systematic way to assess bias in studies examining consumer-grade and research-grade wearable devices. This is the first tool designed to evaluate the risk of bias in validation and reliability studies. Other risk of bias tools like the Cochrane ROB, COSMIN, or the many other risk of bias tools are designed for different study methodologies or are overly broad and largely unnecessary for the specifics of validity/reliability testing studies. The development of the WEAR-BOT involved extensive collaboration among experts, encompassing iterative, open-ended discussions, several rounds of anonymous Delphi-style questionnaires, and pilot testing. The tool comprises detailed checklists for both validity and reliability studies, with subdivisions focusing on study design, methodology, statistical analysis methods, and other critical aspects. The tool balances the need for rigor with ease-of-use. It incorporates a variety of questions to rigorously evaluate the risk of bias in these studies and aims to enhance and standardize methodological approaches in the field. The tool is practical, easily available, and easy to use, as it is built in Microsoft Excel and contains macros that are intuitive and easy to use that allow the user to work more efficiently. The WEAR-BOT represents a significant advancement in the standardization of research methods and statistical analysis in the domain of wearable technology.

## Introduction

The popularity of wearable technology has led to corresponding increases in research on the reliability and validity of these devices. For instance, using the search terms “wearable technology or fitness tracker or activity monitor + validity or reliability” in Google Scholar (Alphabet Inc., Mountain View, CA, USA) produces 486, 1080, 3,640, 11,100, and 14,900 results for the years 2005, 2010, 2015, 2020, and 2022, respectively. There has been a steady increase in this type of research, with a nearly 1000% increase from 2010 to 2020. This nascent technology is being used by a range of different people for numerous use cases, frequently without a clear indication of the validity or reliability of the devices. Individual users, organizations, and even researchers often assume that the objective data on these devices are valid and reliable, frequently without any evidence to support such conclusions. These devices may be used to make training decisions or health assessments, though they may have provided poor results from inaccurate devices. Independent research determines the validity and reliability of these devices so users may be aware of their accuracy under different use cases is warranted. This is especially important as consumer-grade devices and many “research-grade” devices are not regulated by any governing entity. It is our opinion that the thresholds should be tailored to the potential use-case of the devices, and that validity and reliability thresholds for clinical devices should be more stringent than those used for recreational purposes. Additionally, field-specific thresholds may be adopted based on the organization’s preferences, such as collegiate/professional athletics, military/first responders, industrial/construction workers, etc. While the methodologies of testing, analysis, and reporting can be standardized (as we have attempted to do with this tool), thresholds may vary according to device and potential use-case.

The responsibility of testing the accuracy of these devices’ rests upon independent researchers (other than the testing specific organizations may do internally, which are not generally made available to the public). As researchers have sought to validate these devices, the studies have used varied methodologies, statistical analyses, and reporting practices across studies and researchers [1–5]. Methodological and statistical best practices have been suggested by some researchers and organizations, including the Consumer Technology Association and the INTERLIVE Network [1,2,6–13]. These help to guide researchers and manufacturers looking to develop validation studies, although their widespread adoption and knowledge of such standards is still developing among users and researchers (at the time of publication). Nevertheless, these tools and recommendations, as well as those still to come, serve as valuable resources in guiding researchers to perform high quality research. The risk of bias tool introduced below is meant to work in conjunction with, rather than replace any such tools and/or recommendations, as well as add insights and guidance to validation studies that we believe are necessary. The previously published standards and recommendations are not meant to evaluate risk of bias, as the current tool is. Thus, this work represents a unique addition to the previous work that has been done by experts and researchers in this field.

Risk of bias refers to systematic errors that can distort research findings and lead to invalid or inappropriate conclusions. It encompasses several categories, each representing a distinct threat to the validity and/or reliability of a study. Reporting bias involves the selective presentation or omission of outcomes, often leading to an incomplete or skewed interpretation of results. Methodological bias arises from flaws in study protocols, such as inadequate control of confounding variables or poor sampling strategies. Analysis bias, which occurs during the data analysis phase, can arise from inappropriate statistical methods, selective reporting of significant results (e.g., p-hacking), or overfitting models to capture noise rather than true relationships. Other notable types include selection bias, which occurs when the study sample is not representative of the population due to improper recruitment methods, and measurement bias, stemming from the use of invalid or unreliable tools or inconsistent data collection procedures. Each of these biases can undermine the credibility of findings and conclusions drawn by researchers. The questions within the WEAR-BOT are aimed at reducing or evaluating the presence of these potential biases. By evaluating, and thereby encouraging, specific study methodologies, reporting practices, analytic practices, and other aspects associated with validation and reliability testing, we may not be able to eliminate all instances or types of bias, but it will help to reduce or eliminate many aspects of bias that can arise in validity and reliability studies.

Risk of bias assessment tools have several use cases. For instance, they are frequently used (i) when performing systematic reviews to evaluate the literature being reviewed, or (ii) by journal reviewers to assess the bias risk in a particular study, or (iii) by researchers aiming to design a study that will reduce the risk of bias in their own research. There are many tools that have been developed for almost all study designs. Common tools are the Cochrane Risk of Bias (ROB) 2.0 [14], the Cochrane Risk Of Bias In Non-randomised Studies-of Interventions (ROBINS-I) [15], JBI’s critical Appraisal Tools [16–19], along with many others. For assessing the risk of bias and methodological quality for studies on measurement properties, the COnsensus-based Standards for the selection of health status Measurement Instruments (COSMIN) checklist was published in 2006 with additional publications regarding clarifications and guidelines being published subsequently [20–23]. The COSMIN has checklists designed to evaluate measurement properties associated with patient-reported outcome measures (PROMs) and thus spends time on aspects that may not be relevant to validity and reliability literature broadly, or with consumer-grade wearable technology. Their checklist specifically for studies on reliability or measurement error shares many similarities with the WEAR-BOT, though we offer our additional analyses with checklists specifically for validity or reliability that allow for a more comprehensive analysis [24]. The COSMIN has been utilized by researchers performing a risk of bias assessment in conjunction with a systematic review and meta-analysis. Since the initial and subsequent publications of the COSMIN checklists, the field of wearable technology has grown rapidly, and recommended practices have been further established for validity and reliability studies. An updated risk of bias checklist is needed that reflects the many changes that have occurred in wearable technology and make recommendations based on updated best practices for methodology, analysis, and reporting practices. For the convenience of the reader, a summary table comparing some of the different risk of bias tools can be found in the supporting information (S1 Table). As each risk of bias tool is tailored for a specific type of study methodology, there is often not much overlap between the tools. This is why so many have been developed, because there is such a range of study methodologies. Additionally, there are multiple organizations that publish risk of bias tools for study methodologies that already have published tools, as each tool has a slightly different focus on breadth, depth, design, analysis, reporting, or otherwise.

Therefore, the purpose of this paper is to introduce a new risk of bias assessment tool, specifically for assessing methodological quality and the risk of bias in validity and reliability studies using wearable technology, with a focus on consumer-grade wearable technology. While this tool was designed with a focus on consumer-grade wearable technology, it may be applied to all wearable technology. Though medical and clinical devices are regulated by a governing body such as the FDA, users should find similarity between the requirements for clinical approval and the tool provided. The **WEA**rable Technology **R**isk of **B**ias and **O**bjectivity **T**ool (WEAR-BOT) is introduced here, with accompanying instructions for use below.

## Tool development methodology

This tool was developed through iterative discussions and subsequent use with academics and professionals involved in the field of wearable technology, with a focus on testing the validity and reliability of consumer-grade devices. Eight researchers were invited to participate in the development of this tool, and seven agreed to participate. Discussions with individual researchers and the primary investigators were first conducted to introduce the general idea and discuss the need for development of a novel risk of bias tool. Regular group meetings then commenced, spanning several months, where published literature was reviewed and discussed and suggestions for tool development were made. This included reviewing published risk of bias tools, validity and reliability studies, and other recommendations made in published literature, while gradually developing the checklist. Discussion on recommendations, questions, wording, and the overall scope were constantly evaluated over the months of tool development. As researchers were located throughout the United States, all meetings were completed virtually. All meetings were an open-ended discussion, where each researcher contributed as they wished. Decisions on scope, content, wording, and all other aspects of the tool were discussed in an open manner until all researchers were satisfied with the initial results.

Once the questions were mostly established, a pilot study or proof-of-concept systematic review was performed. The results of this systematic review will be published elsewhere. After the completion of the review, any suggestions for changes to the tool from the researchers involved in the systematic review were considered by the entire group and instituted where consensus was reached. After several iterations of the tool were developed, criticized, and pilot tested, several rounds of an anonymous, Delphi-style questionnaire were conducted to determine if consensus had been reached on several aspects of the checklist. These included aspects such as: question wording, category scope, and other aspects. Minor changes were made as a result of the Delphi questionnaires and are reflected in the current version of the WEAR-BOT checklist. Consensus was reached on all aspects of the tool that were questioned in the Delphi questionnaire process.

The final tool can be accessed with the link below. The tool is available for use with Microsoft Excel (Microsoft Corporation, Redmond WA, USA) and contains macros specific Excel that do not transfer over to other spreadsheet software. The link for tool use is: https://doi.org/10.7910/DVN/VJ9D2X.

## Results

**Fig 1:**
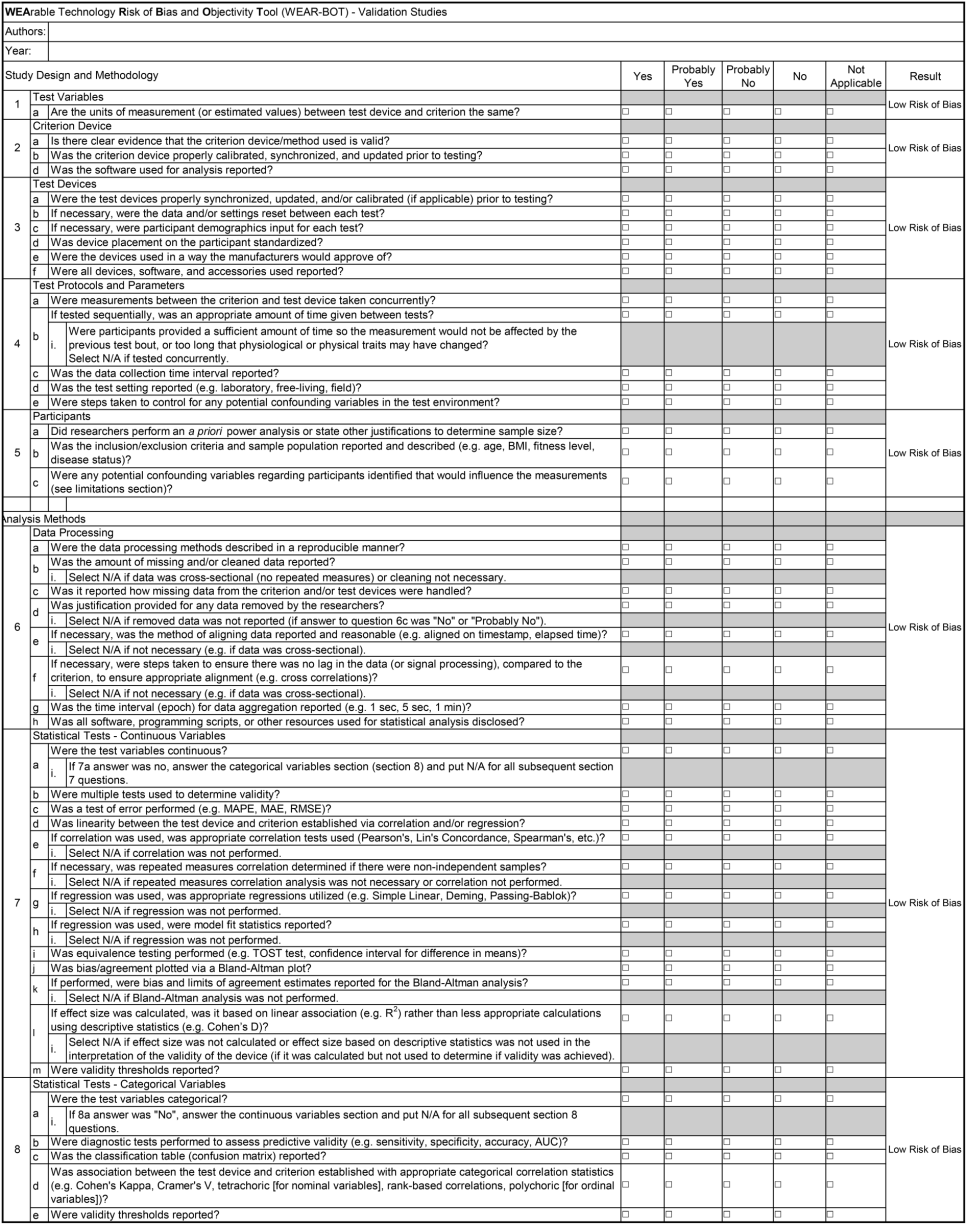
WEAR-BOT checklist for validity studies.

**Fig 2:**
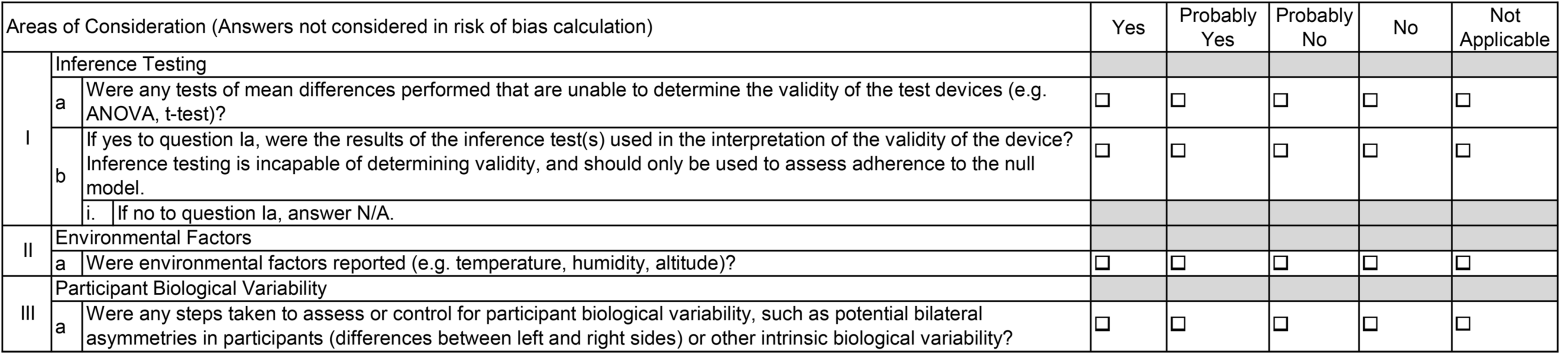
WEAR-BOT Areas of Consideration checklist for validity studies.

**Fig 3:**
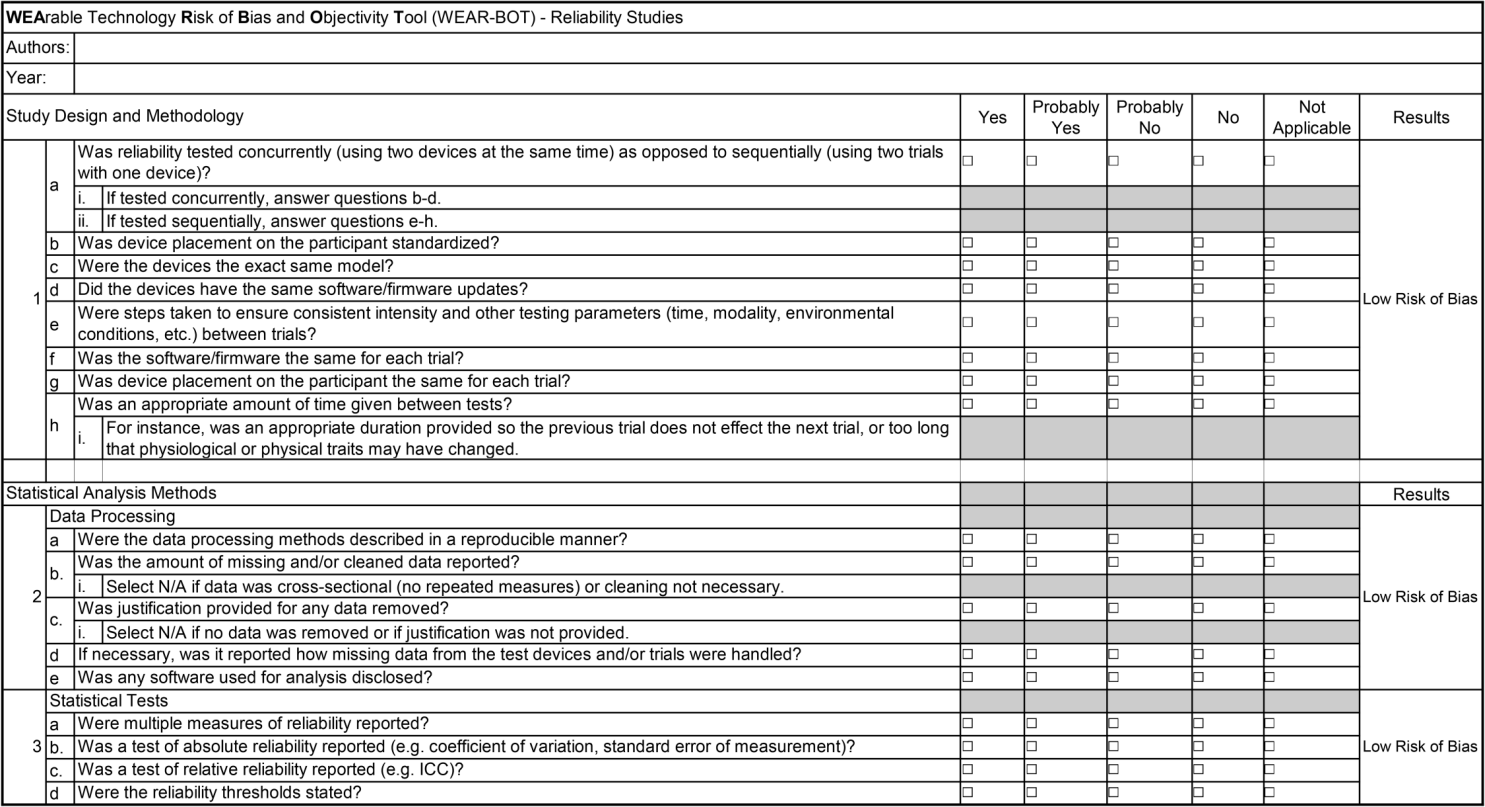
WEAR-BOT checklist for reliability studies.

The novel WEAR-BOT tool consists of two checklists, one for validity studies, and one for reliability studies. Each checklist is split into two broad categories, “Study Design and Methodology”, and “Statistical Analysis Methods”. There are subcategories, that each have questions intended to evaluate the risk of bias found in the study being evaluated. The researcher using the tool must answer one of the following, “Yes”, “Probably Yes”, “Probably No”, “No”, or “Not Applicable”. The subcategories for the validity checklist are 1. Test Variables, 2. Criterion Device, 3. Test Devices, 4. Test Protocols and Parameters, 5. Participants, 6. Data Processing, 7. Statistical Tests – Continuous Variables, and 8. Statistical Tests – Categorical Variables. Subcategories 1–5 are under “Study Design and Methodology”, whereas 6–8 are under “Statistical Analysis Methods” (see Fig 1). The validity checklist also has an “Areas of Consideration”, that the answers are not factored into the overall risk of bias calculations but may be considered by researchers looking to design their own studies (see Fig 2). The reliability checklist has only two subcategories, which are both under “Statistical Analysis Methods”. Therefore, the reliability checklist contains questions for “Study Design and Methodology” (no subcategories), and “Data Processing” and “Statistical Tests” under “Statistical Analysis Methods” (see Fig 3). Some instructions for use are published in the tables/tool itself, for guidance on common issues researchers may run into. For more complete instructions, see below under the “General Guidelines and Instructions for Use” section of this paper.

## General guidelines and instructions for use

This tool is intended for researchers to evaluate previously published research and the risk of bias they may contain, as it relates to validity and reliability studies using consumer-grade wearable technology. This is frequently performed as part of a systematic review. However, researchers looking to perform their own validity or reliability study may also use the checklist to ensure they are designing studies that have a minimal risk of bias. The checklist is designed to reduce bias and improve the validity of the studies, including internal and external validity, by ensuring appropriate study design and methodology, as well as statistical analysis. It can be utilized by researchers evaluating wearable technology concurrently or sequentially, thus improving the evaluation of the test devices concurrent, predictive, and/or criterion validity.

It is important to note that this tool is meant to be both simple to use, and broadly applicable. As such, exact recommendations for every protocol are not included, as those can be seen in Consumer Technology Association Standards papers, or the INTERLIVE Network Validation Protocols [9–13]. Thus, we rely, in part, on the expertise of those seeking to use this tool to evaluate published literature. Several instances of “appropriate” or some version of judging appropriateness can be found in the wording of the questions. As devices and recommendations continue to evolve, we hope the researchers utilizing this tool will use their experience to apply their best judgment when evaluating the literature with this tool.

The following section will provide general instructions for use, describing the overall intent of each section and some general guidelines. Detailed instructions for use can be found below the general guidelines, which will address specific questions researchers may have when utilizing the tool.

### Validity checklist

#### Study design and methodology.

Subsection 1: Test Variables

This section focuses on the alignment of measurement units between the test device and the criterion, ensuring that the validity of the device is assessed against intended measures. It emphasizes the importance of appropriate study design in validation research, discouraging the testing of variables not targeted by the device manufacturers.

Subsection 2: Criterion Device

This section evaluates the selection and utilization of criterion devices, requiring evidence of validity, proper calibration, and detailed reporting on the software used for analysis. This section highlights the need for clear justification when non-standard criterion methods are employed, ensuring the criterion’s relevance and validity.

Subsection 3: Test Devices

This section addresses the standardized use and reporting of test devices, including their calibration, reset procedures, input of participant demographics, and placement on participants. It aims to establish control over potential confounding variables while balancing internal and external validity, ensuring that devices are used as intended by their manufacturers.

Subsection 4: Test Protocols and Parameters

This section emphasizes the importance of controlling all appropriate factors, especially when the criterion measure and test device are not tested concurrently, but are tested sequentially. It is important to control for all possible testing parameters, where appropriate. It recommends the reporting of data collection intervals, test settings, and measures taken to control potential confounders, ensuring thorough evaluation of the test environment.

Subsection 5: Participants

This section focuses on the justification of sample size through power analysis or other means, the reporting of inclusion/exclusion criteria, and the description of sample demographics. Additionally, it calls for the identification of potential confounding variables related to participants that could influence measurements.

#### Statistical analysis methods.

Subsection 6: Data Processing

This section is concerned with the transparency and reproducibility of data processing methods, reporting on missing or cleaned data, and the alignment of data from test devices with the criterion. It stresses the importance of clear methodology in data handling and processing to ensure accurate comparisons in validity studies.

Subsection 7: Statistical Tests – Continuous Variables

This section is the longest section and evaluates the appropriateness of statistical tests for continuous variables, recommending specific tests for 3 different aspects of validity, 1. error, 2. linearity, and 3. equivalence testing, while also recommending a Bland-Altman plot be generated to visually represent measurement bias. It guides the choice of tests based on data characteristics and emphasizes the importance of reporting validity thresholds and proper effect size calculations.

Subsection 8: Statistical Tests – Categorical Variables

This section ensures the use of appropriate diagnostic tests for categorical variables, such as sensitivity, specificity, and accuracy, and the reporting of classification tables. It suggests association tests suitable for nominal or ordinal variables and stresses the importance of establishing and reporting validity thresholds.

#### Areas of consideration.

Finally, this tool mentions additional factors that, while not directly contributing to the risk of bias calculation, may prove valuable for researchers to consider when designing their studies. These considerations are meant to further mitigate bias and enhance the validity of research involving wearable technology.

### Reliability checklist

#### Study design and methodology.

This section prompts researchers to detail their approach to reliability testing, distinguishing between concurrent and sequential methodologies. It emphasizes the importance of standardizing device placement, ensuring device and software uniformity, and maintaining consistent testing parameters across trials. These elements are crucial for minimizing variability and bias, thus enhancing the reliability of study findings.

#### Statistical analysis methods.

Subsection 2: Data Processing

Within this section, the tool addresses the handling and processing of data, including the description of data cleaning methods, reporting of missing or cleaned data, and the justification for any data exclusion. This ensures that the data analysis process is transparent and reproducible. Additionally, it queries the use of specific software for analysis, promoting methodological integrity.

Subsection 3: Statistical Tests

This section encourages the reporting of multiple measures of reliability, differentiating between absolute reliability measures, such as the coefficient of variation and standard error of measurement, and relative reliability, typically assessed using the Intraclass Correlation Coefficient (ICC). This approach to reliability testing provides a thorough understanding of a device’s performance, producing a more robust understanding of the device’s reliability.

### Scoring

The scoring that is performed within the tool is a simple summed amount, based on how many questions were answered “Yes” or “Probably Yes” that would introduce bias, or vice versa for “No” or “Probably No”. To achieve a “Low Risk of Bias”, all questions must be answered in a manner that mitigates or reduces bias. If any of the questions are answered in a way that would introduce bias to the study, the tool will generate “Some Risk of Bias” as the result. To achieve a “High Risk of Bias”, multiple questions must be answered in a way that would introduce bias. The number of questions that generates a “High Risk of Bias” result is dependent on the number of questions for the category. For instance, “Criterion Device” section will require all three questions to be answered in a way that introduces bias to receive a “High Risk of Bias” result. Compared to “Statistical Tests – Continuous Variables”, where five or more questions (out of the 13 in that category) will need to be answered in a way that introduces bias to achieve a “High Risk of Bias” result.

## Detailed instructions for use

### Validity checklist

#### Study design and methodology.

Subsection 1: Test Variables

Question 1a: Are the units of measurement (or estimated values) between test device and criterion the same?

This question is important to determine whether the validity of the device is being tested, or simply correlation between other variables. It would be inappropriate to test whether the device can measure variables the manufacturers did not intend for it to measure in the context of a validation study. While this could be performed in an exploratory manner, the analysis would be different than in validation studies.

Subsection 2: Criterion Device

Question 2a: Is there clear evidence that the criterion device/method used is valid?

This question is important because there must be clear evidence that the device chosen for the criterion is accurate and/or reliable enough to provide the correct values. There have been several studies that use a “criterion device” that is not widely agreed upon to be accurate and/or reliable enough to be considered a criterion, which introduces bias to the study. Although at times it is clear whether a certain measure is accepted as the gold standard, and should be used as the criterion, this is not always the case. In such instances where researchers are using data collection methods that are not widely accepted as the gold standard, it is especially important that researchers evaluate the “criterion” before use and report it in the paper. For example, body composition testing using a DEXA scanner would be widely accepted as a criterion device, but researchers who utilize bioelectric impedance analysis (BIA) may need to establish the device as an appropriate criterion for their study based on previously published data. As has been noted by previous literature, thresholds for validity and reliability are not widely established [25], nor are thresholds for the criterion devices, so researchers may need to use their best judgement when citing a device as a “criterion”, until threshold for criterion devices can be established.

Question 2b: Was the criterion device properly calibrated, synchronized, and updated prior to testing?

This is an important aspect for researchers to report to ensure that there was not a systematic or random bias in the data due to improper calibration, which may cause the validity and/or reliability measures of the test device to be inaccurate due to methodological errors on the researchers’ part. In addition, appropriate syncing with devices or having updated devices for some participants rather than others may introduce differing results, and thus introduce bias into the study.

Question 2c: Was the software used for analysis reported?

This question simply requires the evaluator to determine whether the software used for analysis within the study was reported, an effort to reduce reporting bias.

This is, in part, a judgement call performed by the evaluator to determine whether it was appropriate. This task, of assessing whether a measure or aspect of a study was appropriate, is used several times in the WEAR-BOT. Given the tool’s design to accommodate a broad range of applications, we rely on those using the tool to be experts in their fields, and to use their best judgment as to whether something was appropriate, based on their experience and current best practices.

Subsection 3: Test Devices

These questions are mainly concerned with ensuring that the use of the test devices was standardized and reported, or justification provided if standardization was deliberately not prioritized.

Question 3a: Were the test devices properly synchronized, updated, and/or calibrated (if applicable) prior to testing?

As with calibration of the criterion device, this step is necessary to complete and report for proper bias evaluation. Calibration and syncing of consumer devices often require pairing to a phone or tablet. The use of GPS calculated time performed by the device often reduces the need for researchers to do much more than identify that the time-zone is correct but is not always so simple. Authors should describe how data alignment or syncing between the test device and the criterion was performed. Additionally, the version of the operating system or firmware used in the test devices should be reported, especially if the system was updated within the study timeframe.

Question 3b: If necessary, were the data and/or settings reset or adjusted between each test?

In many wearable devices, previous data may influence the generation of new estimates. For example, a device may use accelerometer data in conjunction with GPS data to determine the stride length of the user. If this data is not reset between tests, and especially between participants, it represents a potential confounding variable. Therefore, it is recommended to reset the settings between tests, when necessary. However, if the researchers are sure that the device does not use previous data to influence the physiologic or physical estimates, then this step is not needed and should be noted in the manuscript.

Question 3c: If necessary, were participant demographics input for each test?

This is important for similar reasons to question “b”. If the test device participant demographics for their estimates, such as bodyweight being used in the calculation of energy expenditure, then not resetting these settings between tests may alter the algorithms used by the manufacturers and create a confounding variable and ultimately misrepresentation of the validity of the devices. Again, if the researchers are sure that demographics are not used in any calculations by the device, this step is not necessary, but should be justified in the manuscript.

Question 3d: Was device placement on the participant standardized?

The placement of test devices should be used as the manufacturers intended, and researchers should report the anatomical attachment point in the paper. With that being said, it should be noted that many wearable devices are meant to be used by the general population, and most manufacturers allow for a level of variety in how they are used. Researchers should seek placement and use that is in-line with manufacturer recommendations. This point is assessed further in question “3e”.

Question 3e: Were the devices used in a way the manufacturers would approve of?

This question assesses in general terms if the device was used appropriately (rather than specific device placement as evaluated in Question 3d). This may require those assessing the study to use their best judgement and read device manuals if there is a question regarding methodology. Similar to device placement, test devices should be used in the manner for which the device was designed, and authors should note this in the manuscript.

Question 3f: Were all devices, software, and accessories used reported?

Some wearable devices have additional accessories that can be used in conjunction with the base device to improve accuracy or broaden the number of variables it can track/estimate. These should be reported, if not, comparison across studies cannot be done appropriately. Additionally, whatever software or applications were used to collect the data should be reported, whether this be the native application for the device, or a third party application.

Subsection 4: Test Protocols and Parameters

Question 4a: Were measurements between the criterion and test device taken concurrently?

This aspect of testing is important to establish because there are additional factors that need to be controlled for when testing sequentially, including device placement, elapsed time, exercise intensity (if being used during exercise), environmental factors, among many others. While testing sequentially can be used for testing, most consumer-grade devices are relatively inexpensive, and thus testing with two devices concurrently, in addition to the criterion (three devices total), is a realistic possibility, and reduces the risk of introducing confounding variables associated with time and multiple data collections.

Question 4b: If tested sequentially, was an appropriate amount of time given between tests?

As stated above, testing sequentially requires researchers to attempt to control many more variables than if testing concurrently to ensure appropriate internal validity. An important factor is time. This is specified in the tool, with instructions stating, “Were participants provided a sufficient amount of time so the measurement would not be affected by the previous test bout, or too long that physiological or physical traits may have changed?”. The amount of time between tests will vary from study to study, and possibly across testing bouts and modalities within the same study. If testing something that does not require a lot of rest time, such as activities of daily living, or walking, minutes may be enough time between trials. However, if testing an exercise modality that requires larger effort, such as running or circuit training, minutes may not be long enough, and researchers may be looking at having hours to days between trials. In addition, waiting too long, such as weeks to months, could be too long between trials, and a person’s physiology may change based on their training status (if testing exercise modalities). This question answer should be N/A if the devices were tested concurrently, as stated in the tool.

Question 4c: Was the data collection time interval reported?

The length of time the data was collected for (the time of the entire trial) should be reported, whether this was five minutes or five hours. This will be especially important for compiling results from multiple studies, as MAPE from a 5-minute bout of exercise should not be weighted the same as MAPE from a 5-hour bout of exercise when compiling an overall MAPE for devices.

Question 4d: Was the test setting reported (e.g., laboratory, free-living, field)?

This question is asked to determine whether the test setting was reported and what environments the device has been tested in, and under what circumstances the device may be considered valid. Knowing the environment the device was tested in is important for contextualizing the results and understanding the conditions under which the device was evaluated. Whether the measurements were taken in a controlled laboratory environment, during free-living conditions, or in a field setting may influence the use cases of the device, limiting it to certain scenarios. By clearly stating the test setting, researchers enable a deeper understanding of the context in which the device performs as expected or reveals potential limitations, guiding both users and developers in making informed decisions about its practical applications.

Question 4e: Were steps taken to control for any potential confounding variables in the test environment?

Authors should report whether potential confounding factors, specifically in their environment, were present during testing. As testing environments may vary greatly, it will be difficult to develop a questionnaire or checklist to address every potential confounding variable. Therefore, we rely on the expertise of those using the tool when evaluating the literature to use appropriate judgement based on previous literature and their experience to identify any potential issues in the methodology of the study being evaluated.

Subsection 5: Participants

Question 5a: Did researchers perform an *a priori* power analysis or state other justifications to determine sample size?

Performing an *a priori* power analysis is important to ensure appropriate power in the study without wasting time and resources by testing too many participants. Researchers should utilize a correlation-based effect size when calculating the necessary sample size (such as Pearson’s Product Moment Correlation Coefficient), as using effect sizes based on descriptive statistics (such as Cohen’s D) would likely result in far too many participants being tested to achieve appropriate power in the study. If a power analysis was not performed, authors should justify the sample size tested in another manner, otherwise the evaluator can select “No” for this question.

Question 5b: Was the inclusion/exclusion criteria and sample population reported and described (e.g., age, BMI, fitness level, disease status)?

An appropriate reporting of participant demographic characteristics should be provided by the researchers. As the goal of validation and reliability studies are to determine if and when wearable devices can produce accurate results, a critical component is the demographics of the population that the device was tested on. Comparing device performance during stride length for the general population vs. individuals with a musculoskeletal disorder would be improper, thus researchers should report the aspects of their population in a thorough manner.

Question 5c: Were any potential confounding variables regarding participants identified that would influence the measurements (see limitations section)?

Authors should report whether any potential confounding factors, specifically regarding participants, were present during testing. This question relies on the researchers utilizing the tool to use their expertise and their best judgement to identify any potential confounding variables that may be present in the unique participant pool for each specific study they assess. The rationale should be sound, cautiously measured, and be stated in the systematic review or report they produce if they identify any potential confounding variables to ensure transparency. As noted in the question, this can generally be found in the “limitations” section of the paper.

#### Statistical analysis methods.

Subsection 6: Data Processing

This subsection is the first under the broader category of “Statistical Analysis Methods,” emphasizing the critical role of proper data processing in research involving wearable technology. It aims to ensure that data processing methods are thoroughly reported and adhere to standards that allow for reproducibility. These questions collectively aim to ensure the methodological rigor and transparency of data processing in research involving wearable technologies, providing a foundation for the subsequent statistical analysis.

Question 6a: Were the data processing methods described in a reproducible manner?

This question assesses whether the methodology section provides a detailed account of the data processing steps, and could include reporting of specific software tools, versions, and settings used. The aim is to determine if another researcher could replicate the study based on the information provided. Data cleaning is “the process of fixing or removing incorrect, corrupted, incorrectly formatted, duplicate, or incomplete data within a dataset” [9]. Data processing is specific to the device and measurement. If the measurement is taken several times throughout the data collection period (such as heart rate or accelerometry data), there may be missing data, or data that needs to be cleaned. However, if the data is cross-sectional, and only a single value is provided by the device (such as estimated VO_2max_), then data cleaning is not necessary. The researchers should examine whether the methods were detailed enough to accurately reproduce the data processing methods, and whether they believe anything was omitted from the processing methodology. It is important that the researchers using the WEAR-BOT be familiar with at least basic types of data processing that may be required when using wearable technology in research, as specific devices have specific data processing needs.

Question 6b: Was the amount of missing and/or cleaned data reported?

This question is pivotal to ensuring data processing, and specifically cleaning of the data, is done transparently and with integrity. Reporting missing data, and ideally any known reasons, such as participant dropout, device malfunction, etc. should be considered. In a validity study, removing data should be done cautiously, and with good justification to prevent biased results. Researchers should report the volume and reasons for data exclusion, such as outliers, errors in the criterion data, or other reasons. In-text instructions to select N/A applies if the data was cross-sectional or if cleaning was deemed unnecessary due to the nature of the data collection method.

Question 6c: Was it reported how missing data from the criterion and/or test devices were handled?

This question is centered on the methodology for addressing missing data, ensuring that such handling does not bias the results. Reporting for this parameter may be as simple as stating the amount of missing data from each device. The amount of missing data will be relative to the granularity of the epoch (and possibly signal frequency). For example, devices that aggregate sensor data every second compared to every minute will likely have more missing data. If the device outputs raw data (unusual in consumer-grade electronics), the scale of processing and missing data will be much greater. This should be noted in the report and taken into account by the researchers evaluating the study. If imputation was performed, justification must be reported, as in most cases, imputation for validity studies of consumer-grade wearable devices would be inappropriate.

Question 6d: Was justification provided for any data removed by the researchers?

This question seeks to ensure that any decision to exclude data from the analysis is transparent and justified in the report. It underscores the necessity for researchers to provide a clear and well-founded rationale for any data they decide to remove or exclude from their study. Such justifications are crucial for understanding the boundaries and conditions the device being tested can be found valid or not valid. The criteria for data removal can vary widely, from errors in the criterion device (which should be removed so the test device is not faulted due to the criterion’s mistake), to errors in data collection or entry. The explicit reporting of these criteria not only enhances the study’s reproducibility but also allows for a critical assessment of its findings. In-text instructions to select N/A if no data was removed or if the removal was not explicitly reported are present.

Question 6e: If necessary, was the method of aligning data reported and reasonable (e.g., aligned on timestamp, elapsed time)?

The proper alignment of data points is crucial for comparison across devices or time points. This question evaluates whether the study described how data from different sources were synchronized. Options for alignment could be either by timestamps, event markers, or elapsed time. Other methods may be acceptable if the researcher described the process, why it was chosen, and why it was deemed necessary. Ensuring that measurements are properly aligned must be done in order for the validity of the device to be properly tested. Alignment of data, however, may not be necessary for all devices. In the instance that the data is cross-sectional (e.g., total hours of sleep), alignment is not necessary as there are not repeated measures. Therefore, evaluators have in-text instructions to select N/A if alignment was not a necessary step in the data processing phase.

Question 6f: If necessary, were steps taken to ensure there was no lag in the data (or signal processing), compared to the criterion, to ensure appropriate alignment?

This question also deals with data alignment but is a recommended step to ensure any potential lags between devices were identified and corrected. Techniques such as cross-correlation analysis might be employed to verify temporal alignment. Cross correlation is a statistical method used to measure the similarity or correlation between two datasets as a function of the lag of one relative to the other. Essentially, it helps to identify the degree to which two series are correlated at different time shifts, enabling the detection of patterns or relationships that may not be immediately evident in unshifted or simultaneously collected data. If there are significant correlations in the shifted data, an offset should be used to properly align the data to account for the lag present. Again, evaluators are provided in-text instruction to select N/A if cross correlations are not necessary (if the data is cross-sectional).

Question 6g: Was the time interval (epoch) for data aggregation reported (e.g., 1 sec, 5 sec, 1 min)?

This question examines whether the study clearly reported the time intervals or epochs over which data was aggregated and assesses the suitability of these intervals for the study’s objectives. The granularity of the data, or the smallest time unit of aggregation, is crucial as it influences the level of detail captured and the amount of data processing needed. For example, data aggregated at shorter intervals (e.g., every 5 seconds) can capture more detailed variability but may require more extensive cleaning than data aggregated at longer intervals (e.g., every 5 minutes), which would smooth over finer variations. The choice of aggregation interval impacts not only the processing and analysis of data within the study but also the comparability and synthesis of findings across studies and devices, especially in future meta-analyses. Therefore, it’s important that the selected intervals are justified in the context of the study’s goals and the characteristics of the data collected. Each device and measurement may justify different epoch’s. Closely associated with epoch is the signal frequency, which ideally would be reported, if known. However, as consumer devices rarely report raw data, the aggregated data is what we are most concerned about. Sampling frequency may be a necessary factor to account for during certain use-cases that require more accuracy, and as meta-analyses are completed, they may want to address the issue of epoch and signal frequency in any weighted averages for devices. For the purposes of this tool, reporting the epoch is sufficient to reduce bias.

Question 6h: Was all software, programming scripts, or other resources used for statistical analysis disclosed?

Transparency in the tools and software used for analysis is critical for reproducibility. This includes not only the names and versions of statistical packages but also any custom scripts or code developed for the study.

Subsection 7: Statistical Tests – Continuous Variables

This subsection discusses the statistical methodologies applied to continuous variables in validation studies, recommending several statistical tests and reporting methods as best practices. This detailed approach to evaluating the application of statistical tests to continuous variables ensures that the methodologies employed are rigorously scrutinized for appropriateness, comprehensiveness, and transparency, when evaluating the credibility and risk of bias of the validation study.

Question 7a: Were the test variables continuous?

This preliminary question establishes the nature of the data used by researchers, confirming that the subsequent questions are relevant to the study’s statistical analysis. If the test variables are not continuous, the evaluator should go on to subsection 8, which has questions pertaining to categorical variables, and mark subsequent questions in this section as N/A.

Question 7b: Were multiple tests used to determine validity?

This question is important to understand whether a comprehensive approach was taken in analyzing the data, employing multiple statistical tests to evaluate the validity of the findings. This approach is important for a thorough evaluation of wearable technology. While we recommend specific tests later in the tool, this question does not require those specific tests to be performed, and evaluators may select “Yes” or “Probably Yes” if the original researchers utilized multiple tests that they claim to be used for validity analysis.

Question 7c: Was a test of error performed (e.g., MAPE, MAE, RMSE)?

This question asks about error testing, which is among the most common aspects of validity that is currently tested in published works. While we recommend that a test of error is performed, we do not specify which error measurement should be used. While mean absolute percentage error (MAPE) is the most common and makes it easy to compare results across different variables, as percentages are widely understood metrics, some researchers may prefer root mean square error (RMSE) or mean absolute error (MAE) which both will maintain the original units of whatever estimate or calculation the devices use, or other error measurement. Ultimately, the selection of an appropriate error metric should be chosen based on the nature of the data, study protocols, and specific objectives of the study.

Question 7d: Was linearity between the test device and criterion established via correlation and/or regression?

This question addresses the fundamental aspect of establishing a linear relationship between the measurements obtained from the test device and those from the criterion device in validation studies. Linearity is crucial because it indicates that the test device can accurately reflect changes in the variable of interest across the range of measurements in a manner consistent with the criterion device. It is necessary to demonstrate that for any increase or decrease in the measured variable, the test device’s response is directly proportional to that of the criterion device, without systematic overestimation or underestimation at specific ranges. Establishing linearity involves statistical methods such as correlation analysis, which assesses the strength and direction of the relationship between two variables, and regression analysis, which models the relationship between a dependent variable (test device readings) and an independent variable (criterion device readings). A strong linear relationship (e.g., high correlation coefficient, regression line closely fitting the data points) provides evidence that the test device is capable of accurately tracking the criterion across its measurement spectrum. The question of which correlation and regression tests should be used will be specified below in subsequent questions. Additionally, where appropriate, confidence intervals would ideally be reported. Although, as confidence intervals can be calculated from summary statistics, is not strictly necessary.

Question 7e: If correlation was used, was the appropriate correlation test employed (Pearson’s, Lin’s Concordance, Spearman’s, etc.)?

This question examines the selection of correlation tests in the study. We do not recommend a specific correlation test, as different data may require specific tests. However, some considerations as to when you would use each test can be found here. Pearson’s correlation coefficient can be used for continuous variables with a normal distribution, offering straightforward linear relationship insights. It’s widely used, facilitating comparisons across studies and allowing for sample size calculations with common statistical software. Spearman’s rank correlation is suited for non-normally distributed data, as it assesses the relationships through ranking, thus providing a viable option for non-linear associations. Lin’s Concordance Correlation Coefficient, on the other hand, is generally recommended for validation studies due to its comprehensive assessment of both precision and accuracy between two variables. This makes Lin’s particularly valuable when evaluating the agreement between a test device and a criterion standard. The choice between these tests hinge on the data’s distribution and the study’s specific needs. Justification should be provided by the original researchers as to why they used certain tests, and the evaluators best judgement should be used to determine if it was appropriate for the study being evaluated.

Question 7f: If necessary, was repeated measures correlation determined if there were non-independent samples?

This question is important and often overlooked in validation studies, which is why it is further specified from the previous question. The aim of this question is to address the analysis of data from studies with measurements that are not independent, typically seen with repeated measures on the same subjects. Traditional correlation assessments may not accurately depict the relationship between variables due to the interrelated nature of these data points. The use of repeated measures correlation tests, such as the repeated measures correlation or an intraclass correlation coefficient (ICC) designed to handle multiple measurements is necessary to properly evaluate linearity in validation studies with repeated measures.

Question 7g: If regression was used, was the appropriate regressions utilized (e.g., Simple Linear, Deming, Passing-Bablok)?

This question evaluates the tests for linearity, specifically different regression models. Choosing the right type of regression analysis is important to accurately assess the relationship between the measurements obtained from the test device and those from the criterion device. While we do not recommend specific tests in the WEAR-BOT checklist, the reader can find brief explanations of when to use different models for validity testing. Simple linear regression is the most straightforward approach, modeling the relationship between a single independent variable and a dependent variable by fitting a straight line through the data points. This is the most widely known form of regression, and due to its simplicity, the most digestible for the reader. However, it assumes that the independent variable (criterion device measurements) is measured without error, which may not always be the case in validation studies where both devices could have measurement errors. Deming regression, also known as errors-in-variables regression, extends beyond simple linear regression by accounting for measurement errors in both the test and criterion devices. This method adjusts the regression line based on the ratio of the variances of the measurement errors, offering a more accurate estimation of the relationship when both variables have associated uncertainties. Deming regression is generally the preferred model for validity studies where the data is normally distributed. Passing-Bablok regression is a non-parametric approach that, like Deming regression, does not assume one of the variables to be error-free. It is robust against outliers and does not require the distribution of measurement errors to be normal, making it suitable for a wide range of data types. Therefore, Passing-Bablok will be the better regression model if the data is not normally distributed. Taking into account these considerations will enable the researchers to utilize the correct regression model and allow evaluators to properly assess the risk of bias in the statistical methods of the studies in question.

Question 7h: If regression was used, were model-fit statistics reported?

This question evaluates if results were reported from the regression model used. In validation studies where regression analysis is employed to examine the relationship between a test device and a criterion standard, reporting model performance is recommended for a comprehensive understanding of the model’s fit and predictive accuracy. The coefficient of determination (R^2^), residual sum of squares, y-intercept, and slope of the regression line are appropriate for simple linear regression, while Deming and Passing-Bablok regression should report the y-intercept and slope, as they do not produce a true R^2^ value. Together, these metrics provide a detailed account of the linear relationship, allowing for an evaluation of how well the test device’s measurements align with those of the criterion across the range of values tested. Reporting these metrics will allow the reader to better understand the amount of linearity between the devices, and will allow for comparisons between studies in future meta-analyses.

Question 7i: Was equivalence testing performed (e.g., TOST test, confidence interval for difference in means)?

This question investigates whether the study included equivalence testing to statistically determine if the test device’s measurements are acceptably close to those of the criterion device. Equivalence testing, such as the Two One-Sided T-Tests (TOST test) and analysis using confidence intervals for the difference in means, is another method to determine validity (or equivalence) in validation studies. These can be used to establish that the test device’s measurements are statistically equivalent to a criterion standard within a pre-defined margin. Unlike traditional hypothesis tests aiming to find significant differences, equivalence testing flips the null hypothesis and verifies that any deviations between devices are within acceptable limits. The TOST test procedure, for instance, checks if differences fall within specified equivalence bounds, offering a stringent criterion for method validation. This ensures the test device performs closely to the standard, supporting its use for the intended applications with confidence. This is particularly relevant for validation studies aiming to establish that two measurement methods agree within a tolerable margin of error.

Question 7j: Was bias/agreement plotted via a Bland-Altman plot?

This question simply asks if the researchers utilized Bland-Altman plots for assessing agreement/bias between the test device and the criterion. This graphical method is a widely utilized method for identifying any systematic bias and the limits of agreement in validation studies. By providing a visual representation of how the differences between the two measurement methods vary across the range of measurements, Bland-Altman plots facilitate the identification of any systematic bias or trends, such as a tendency for differences to increase as the magnitude of the measurement increases.

Question 7k: If performed, were bias and limits of agreement estimates reported for the Bland-Altman analysis?

This question is to evaluate whether, in addition to plotting, the study reports quantitative estimates of bias (average difference) and limits of agreement as calculated from the Bland-Altman analysis, providing a clear indication of the test device’s accuracy. Reporting this is valuable to the readers and may be used in the future for comparisons between devices or modalities.

Question 7l: If effect size was calculated, was it based on linear association (e.g., R^2^) rather than less appropriate calculations using descriptive statistics (e.g., Cohen’s D)?

This question ensures the appropriate use of effect sizes for studies performing validity testing, such as those based on linear associations, rather than those more suited to comparing group means. Utilizing effect sized based on descriptive statistics (group means) will produce small effect sizes for most validation studies, as the goal of the test device is to be as close to the criterion in its measurements as possible. Therefore, it would be inappropriate to use effect sizes based on descriptive statistics and association-based effect sizes should be utilized. However, if the effect size based on descriptive statistics was not utilized in the interpretation of the validity of the device, that would not introduce bias into the study. Therefore, we include in-text instructions that state, “Select N/A if effect size was not calculated or effect size based on descriptive statistics was not used in the interpretation of the validity of the device (if it was calculated but not used to determine if validity was achieved).”

Question 7m: Were validity thresholds reported?

This question gets to the very heart of validity studies, to answer the question of whether a device was valid or not. As thresholds for validity have not been widely established (as of the publication of this paper), it is left up to the individual researchers to determine whether the device meets their standards. There have been several authors who propose varying thresholds for validity, some more conservative, and others more liberal. Whatever thresholds the researcher chooses should be established prior to data collection and reported in the published work. For researchers looking to establish appropriate thresholds of validity and reliability for specific devices they are interested in testing, we believe that the thresholds should be tailored to the potential use-case of the devices, and that validity and reliability thresholds for clinical/research devices should be more stringent than those used for recreational purposes.

Subsection 8: Statistical Tests – Categorical Variables

This subsection addresses the application and analysis of categorical variables within the context of consumer-grade wearable device validation studies. It emphasizes the importance of selecting appropriate statistical methodologies for analyzing categorical data, ensuring the validity and reliability of the devices under study.

Question 8a: Were the test variables categorical?

This question serves as a preliminary filter, confirming whether the data analyzed in this section is indeed categorical. If the data is not categorical, in-text instructions direct the reader to fill out the previous section on continuous variables and select “Not Applicable (N/A)” for all subsequent section 8 questions.

Question 8b: Were diagnostic tests performed to assess predictive validity (e.g., sensitivity, specificity, accuracy, AUC)?

This question evaluates the use of diagnostic accuracy tests to determine how well the device can correctly classify or predict outcomes compared to a criterion standard. This includes assessing whether measures such as sensitivity (true positive rate), specificity (true negative rate), overall accuracy, and area under the receiver operator curve (AUC) were calculated and reported, providing insight into the device’s performance in categorical terms. This could be used for human activity recognition, where devices are attempting to predict what activity is being performed (e.g., walking, washing dishes), or classifying exercise intensity into light, moderate, and vigorous intensity exercise based on metabolic equivalents (METs), or other categories. Overall, these tests are fundamental in evaluating the predictive validity of a test device in classifying or predicting categories against a criterion standard.

Question 8c: Was the classification table (confusion matrix) reported?

This question simply seeks to confirm that the study provided a confusion matrix, detailing the number of true positives, true negatives, false positives, and false negatives. This matrix is an important reporting metric for understanding the device’s classification accuracy and for calculating the diagnostic tests mentioned in question 8b, as well as being an avenue to improve transparency in the results.

Question 8d: Was association between the test device and criterion established with appropriate categorical correlation statistics (e.g., Cohen’s Kappa, Cramer’s V, tetrachoric [for nominal variables], rank-based correlations, polychoric [for ordinal variables])?

This question attempts to ensure that appropriate association statistics were run for the categorical variables, as correlation tests for continuous variables (such as Pearson’s) are inappropriate to use for categorical variables. In validation studies, establishing the association between the test device and the criterion standard requires selecting the appropriate correlation statistics tailored to the data’s nature. Cohen’s Kappa is a robust measure used to assess the agreement between two raters or methods categorizing data into nominal categories, correcting for chance agreement. It is particularly useful when the categories are mutually exclusive and exhaustive. Cramer’s V expands this concept to cases with more than two categories, providing a measure of association between nominal variables. For data that fall into ordered categories, rank-based correlations like Spearman’s rho can be applied to assess the relationship between two variables. When the data is dichotomous or ordinal but assumed to follow an underlying continuous distribution, tetrachoric (for dichotomous variables) and polychoric (for ordinal variables) correlations are preferred as they estimate the Pearson correlation coefficient that would have been obtained if the underlying continuous variables were observed. These statistics can be used in validation studies for assessing the strength of the association between categorical outcomes measured by the test device and the criterion, ensuring that the chosen method aligns with the data’s characteristics and the study’s objectives.

Question 8e: Were validity thresholds reported?

As stated previously in question 7n, this question gets to the very heart of validity studies, to answer the question of whether a device was valid or not. As thresholds for validity have not been widely established (as of the publication of this paper), it is left up to the individual researchers to determine whether the device meets their standards. There have been several authors who propose varying thresholds for validity, some more conservative, and others more liberal. Whatever thresholds the researcher chooses should be established prior to data collection and reported in the published work. Developing thresholds for categorical variables may pose more challenges than for continuous variables, and there have been few thresholds suggested based on diagnostic tests. Fortunately for evaluators, there is no judgement call to be made whether the researchers’ thresholds were appropriate, but rather whether they were reported. As widely accepted thresholds are developed, this tool may need to change to reflect the updated practices.

#### Areas of consideration.

This section addresses additional factors that, while not directly affecting the risk of bias score, may provide context for interpreting the validity and reliability of wearable device studies. These considerations encompass inference testing, environmental factors, and participant biological variability, offering insights into the risk of bias of the studies. These areas were of concern to many of the authors, but consensus was not able to be reached by the entire group to include it into the risk of bias tool calculations, therefore we offer it here, as areas of consideration.

I. Inference Testing

Question Ia: Were any tests of mean differences performed that are unable to determine the validity of the test devices (e.g., ANOVA, t-test)?

This question probes the use of inferential statistical tests designed to compare group means, such as ANOVA or t-tests, which are not directly applicable for validating a device’s measurements. This does not apply to tests that utilize inference tests in a manner specifically designed for validity testing — such as the TOST test — which is a type of equivalence test. Simply interpreting a significant or non-significant p-value is inappropriate for determining validity.

Question Ib: If yes to question Ia, were the results of the inference test(s) used in the interpretation of the validity of the device?

This question inquires whether the outcomes of these hypothesis tests were used to draw conclusions about the device’s validity, despite their inherent limitations for this purpose. This question highlights the importance of distinguishing between statistical significance and practical relevance in the context of device validation. Simply performing these tests (maybe because a reviewer requested it) will not inherently increase bias in the paper, but using them in the interpretation of the validity of the device is surely a poor choice.

II. Environmental Factors

Question IIa: Were environmental factors reported (e.g., temperature, humidity, altitude)?

This question assesses the reporting of environmental conditions during data collection, recognizing their potential impact on the performance of wearable devices. Detailed reporting of such factors may help identify possible confounding variables that could influence the validity of the test device under certain circumstances. In any case, understanding the conditions under which the device was validated can only improve the strength of the paper guide future generalization of the results.

III. Participant Biological Variability

Question IIIa: Were any steps taken to assess or control for participant biological variability, such as potential bilateral asymmetries in participants (differences between left and right sides) or other intrinsic biological variability?

This question is meant to evaluate whether the study being evaluated accounted for biological variability among participants that could potentially affect the measurement accuracy of the device. Due to the variability inherent in this aspect, recommendations for best practices in validity studies would be difficult to establish. Therefore, it remains in the “Areas of Consideration”, and if further research establishes how to deal with participant biological variability, this tool may need to be updated to reflect the recommendations of researchers.

### Reliability checklist

#### Study design and methodology.

Question 1a: Was reliability tested concurrently (using two devices at the same time) as opposed to sequentially (using two trials with one device)?

This question determines which approach to reliability testing was used, concurrent or sequential. Concurrent testing assesses reliability by using two devices simultaneously on a subject, whereas sequential testing uses the same device across multiple trials. This distinction is necessary for understanding the context in which reliability is assessed and directs the subsequent focus of the evaluation. Evaluators should answer “Yes” if there is clear evidence that the devices being tested concurrently were the exact same model (i.e., it is explicitly stated in the manuscript) or answer “Probably Yes” if there are indications the devices were the exact same model but it was not explicitly stated in the manuscript.

In-text instructions direct evaluators to answer specific questions, based on which testing methodology was used. Concurrent testing methodologies answer questions b-d, while sequential testing methodologies answer question b, and e-h.

Question 1b: Was device placement on the participant standardized?

This question checks to see if the placement of devices during testing was consistent and according to approved manufacturer protocols, ensuring that data comparability is not compromised by variations in device positioning. As stated earlier, these devices should be used as the manufacturers designed them to be used, and to ensure that they were, researchers should report how they were used in their papers. With that being said, it should be noted that these devices are meant to be used by the general population, and most manufacturers allow for some level of variety in how they are used. As long as the placement and use are reported and in-line with manufacturer recommendations, this question should be answered “Yes”. However, device placement standardization is particularly important for reliability testing, because as many variables as possible need to be the same between trials, or for each device. Therefore, device placement must be standardized and appropriate in reliability studies.

Question 1c: Were the devices the exact same model?

This question confirms that the devices used for concurrent testing were of the same make and model, eliminating variability that could arise from hardware differences. If the devices were not the exact same make and model, then that introduces serious bias into the reliability study.

Question 1d: Did the devices have the same software/firmware updates?

This question verifies that both devices were operating on the same software or firmware version, ensuring that any differences observed are not due to discrepancies in software functionality. As with many of the questions for the reliability testing section, researchers need to ensure that as many variables as possible are consistent between devices when testing concurrently.

Question 1e: Were steps taken to ensure consistent intensity and other testing parameters (time, modality, environmental conditions, etc.) between trials?

This question examines whether measures were in place to maintain uniform testing conditions across trials, such as exercise intensity, environmental conditions, activity modality, among other testing parameters, to ensure the reliability of results. As the testing environment, activity, and other aspects of the reliability studies can vary widely, evaluators must use their best judgement to decide whether enough effort was taken to control for potential confounding variables to ensure successful tests.

Question 1f: Was the software/firmware the same for each trial?

This question ensures that no software or firmware updates occurred between trials that could influence the comparability of data collected sequentially. This is particularly important if a significant amount of time has passed since the initial trial.

Question 1g: Was device placement on the participant the same for each trial?

This question checks to see if the placement of device during testing was consistent between trials and according to approved manufacturer protocols, ensuring that data comparability is not compromised by variations in device positioning. As stated earlier, these devices should be used as the manufacturers designed them to be used, and to ensure that they were, researchers should report how they were used in their papers. With that being said, it should be noted that these devices are meant to be used by the general population, and most manufacturers allow for some level of variety in how they are used. As long as the placement and use are properly reported and in-line with manufacturer recommendations, this question should be answered “Yes”. However, device placement standardization is particularly important for reliability testing, because as many variables as possible need to be the same between trials, or for each device. Therefore, device placement must be standardized and appropriate in reliability studies.

Question 1h: Was an appropriate amount of time given between tests?

This question examines the scheduling of sequential trials within a study, assessing whether the interval between them was appropriately chosen to mitigate carryover effects from prior activities while also being close enough to prevent any significant alterations in participants’ physiological or physical states. The optimal timing between tests is vital to ensure that each measurement reflects the intended conditions without interference from previous tests or natural variations in participants’ health or performance over time. Evaluators must consider several factors, including the intensity of any exercise or activity involved, the total time commitment required from participants, and the nature of the measurements being taken, among other potential variables. For example, high-intensity activities may necessitate longer recovery periods to return to baseline conditions, while assessments of more stable physiological markers might allow for shorter intervals. The appropriateness of the time interval is thus contingent upon a nuanced understanding of the study’s design and objectives, requiring evaluators to apply their expertise and knowledge of the field to determine whether the chosen intervals were suitable for the study’s aims.

#### Statistical analysis methods.

Subsection 2: Data Processing

Question 2a: Were the data processing methods described in a reproducible manner?

This question assesses whether the methodology section provides a detailed account of the data processing steps, and could include reporting of specific software tools, versions, and settings used. The aim is to determine if another researcher could replicate the study based on the information provided. Data cleaning is “the process of fixing or removing incorrect, corrupted, incorrectly formatted, duplicate, or incomplete data within a dataset” [9]. Data processing is specific to the device and measurement. If the measurement is taken several times throughout the data collection period (such as heart rate or accelerometry data), there may be missing data, or data that needs to be cleaned. However, if the data is cross-sectional, and only a single value is provided by the device (such as estimated VO_2max_), then data cleaning is not necessary. The researchers should examine whether enough data was provided to accurately reproduce the data processing methods, and whether they believe anything was omitted from the processing methodology. It is important that the researchers using the WEAR-BOT be familiar with at least basic types of data processing that may be required when using wearable technology in research, as specific devices have specific data processing needs.

Question 2b: Was the amount of missing and/or cleaned data reported?

This question is pivotal to ensuring data processing, and specifically cleaning of the data, is done transparently and with integrity. Reporting missing data, and ideally any known reasons, such as participant dropout, device malfunction, etc. should be considered. In a reliability study, removing data should be done cautiously, and with good justification to prevent biased results. Researchers should report the volume and reasons for data exclusion, such as outliers, errors in the criterion data, or other reasons. In-text instructions to select N/A applies if the data was cross-sectional or if cleaning was deemed unnecessary due to the nature of the data collection method.

Question 2c: Was justification provided for any data removed?

This question seeks to ensure that any decision to exclude data from the analysis is transparent and justified in the report. It underscores the necessity for researchers to provide a clear and well-founded rationale for any data they decide to remove or exclude from their study. Such justifications are crucial for understanding the boundaries and conditions the device being tested can be found reliable or not reliable. The criteria for data removal can vary widely, but the explicit reporting of these criteria not only enhances the study’s reproducibility but also allows for a critical assessment of its findings. In-text instructions to select N/A if no data was removed or if the removal was not explicitly reported are present.

Question 2d: If necessary, was it reported how missing data from the test devices and/or trials were handled?

This question probes the transparency and methodological rigor with which a study addresses the possible issue of missing data, a common challenge in research involving wearable technology. It assesses whether the researchers provided a clear account of the approaches used to manage gaps in the data, which could range from sophisticated imputation techniques that estimate missing values based on available information to straightforward exclusion criteria that remove incomplete observations from the analysis. The chosen strategy for handling missing data is pivotal, as it can significantly influence the study’s findings and their reliability. Imputation is generally not recommended in reliability studies examining consumer-grade wearable technology, so if it was performed, good justification must be provided. By detailing these methods, a study ensures that other researchers can accurately replicate the analysis and assess the robustness of the conclusions drawn, thereby enhancing the credibility of the research.

Question 2e: Was any software used for analysis disclosed?

This question simply checks if the study provided detailed information on the software tools and versions used for data analysis, promoting transparency and reproducibility. Not doing so would introduce a risk of bias.

Subsection 3: Statistical Tests

Question 3a: Were multiple measures of reliability reported?

This question scrutinizes the depth of the reliability analysis conducted in the study by inquiring if a range of statistical measures were utilized to assess the reliability of a device. This question does not recommend which tests should be performed, but is to ensure a complete assessment of the reliability was performed. A singular measure, while informative, might not fully capture the nuances of a device’s performance across different conditions and metrics. For example, reporting both the Intraclass Correlation Coefficient (ICC) for relative reliability and the Standard Error of Measurement (SEM) for absolute reliability provides a more rounded view of the device’s consistency and the precision of individual scores, respectively.

Question 3b: Was a test of absolute reliability reported (e.g., coefficient of variation, standard error of measurement)?

This question delves into whether the study reported measures of absolute reliability. Some examples of tests being the coefficient of variation (CV) and the standard error of measurement (SEM). Absolute reliability refers to the degree to which repeated measurements vary for individuals, emphasizing the importance of understanding the inherent measurement error and its impact on the precision of the device. The SEM provides a direct measure of this error in the same units as the measurements themselves, offering a clear indication of the expected range within which a measurement might vary due to random error. A device that is perfectly reliable would have a SEM of 0. On the other hand, the CV is the ratio of the standard deviation to the mean, and expresses the reliability of a device as a percentage, providing an easily understandable metric of reliability that can be used across measurements without the need for conversions. These tests provide context of the precision of the device in question. By reporting these measures of absolute reliability, researchers provide the readers with the necessary information to assess the reliability of the device they are testing.

Question 3c: Was a test of relative reliability reported (e.g., ICC)?

This question simply assesses whether the study reported a measure of relative reliability, possibly through the use of the Intraclass Correlation Coefficient (ICC). Relative reliability refers to the degree to which individuals maintain their position in a sample over repeated measurements under varying conditions, highlighting the consistency and reproducibility of the measurements. The ICC is a versatile statistical tool used to evaluate this aspect of reliability, offering a quantifiable measure of the correlation between measurements taken at different times or under different conditions. By reporting a measure of relative reliability, researchers provide the readers with necessary information to assess the reliability of the device they are testing.

Question 3d: Were the reliability thresholds stated?

This question gets to the very heart of reliability studies, to answer the question of whether a device was reliable or not. As thresholds for reliability have not been widely established (as of the publication of this paper), it is left up to the individual researchers to determine whether the device meets their standards. There have been several authors who propose varying thresholds for reliability, some more conservative, and others more liberal. Whatever thresholds the researcher chooses should be established prior to data collection and reported in the published work.

## Supporting information

S1 Table Comparison table between common risk of bias analysis tools. (DOCX)
