# Correction: The WEAR-BOT checklist: A risk of bias tool for evaluating validity and reliability research in wearable technology

**DOI:** 10.1371/journal.pone.0345217

**Published:** 2026-03-17

**Authors:** Bryson Carrier, Jennifer A. Bunn, Chris Eschbach, Joel D. Reece, Gregory J. Welk, Brett A. Dolezal, James W. Navalta

The Funding statement for this article is incorrect. The correct Funding statement is as follows: Publication was supported by a University of Nevada, Las Vegas Libraries Open Article Fund award awarded to J.N. The specific roles of this author are articulated in the ‘author contributions’ section. The funders had no role in study design, data collection and analysis, decision to publish, or preparation of the manuscript.

## References

[1] Carrier B, Bunn JA, Eschbach C, Reece JD, Welk GJ, Dolezal BA, et al. The WEAR-BOT checklist: A risk of bias tool for evaluating validity and reliability research in wearable technology. PLoS One. 2026;21(2):e0338014. doi: 10.1371/journal.pone.0338014 41662393 PMC12885281

