# Supplementary material for: The WEAR-BOT checklist: A risk of bias tool for evaluating validity and reliability research in wearable technology
Source: PLoS One. 2026 Feb 9;21(2):e0338014. doi: 10.1371/journal.pone.0338014 (PMC12885281; doi:10.1371/journal.pone.0338014)
Supplement: S1 Table — (DOCX) [file pone.0338014.s001.docx]

| Topic | ROB 2 | ROBINS-I | COSMIN PROMs | COSMIN Reliability | JBI DTA | WEAR-BOT |
| --- | --- | --- | --- | --- | --- | --- |
| Study design addressed | Randomized trials | Nonrandomized interventions | PROM measurement properties | Reliability and measurement error studies | Diagnostic accuracy studies | Wearable device validity and reliability |
| Randomization process and allocation concealment | Covered | Not applicable | Not applicable | Not applicable | Not applicable | Not applicable |
| Confounding and comparability of groups | Implicit via randomization checks | Covered explicitly | Not a focus | Not a focus | Handled via sampling and design items | Addresses design choices and participant factors |
| Deviations from intended interventions | Covered | Covered, ITT and per-protocol variants | Not a focus | Not a focus | Not applicable | Not a focus |
| Missing data | Covered | Covered | Implicit within boxes when relevant | Implicit within standards | Covered via analysis completeness | Covered in data processing |
| Outcome measurement, assessor blinding, and measurement validity | Covered | Covered | Covered as measurement properties | Covered | Covered for index and reference tests | Covered |
| Selective reporting of outcomes and analyses | Covered | Covered | Not a primary focus | Not a primary focus | Not explicit | Addresses reporting and analysis bias |
| Sampling frame and participant selection | Baseline balance checks | Selection into study domain | Target population and scope specified | Population specified by study aim | Patient selection items covered | Participant inclusion and sample size justification |
| Test procedures and timing | Trial protocol focus | Protocol adherence focus | Instrument development and testing procedures | Repeated measures design details | Index and reference test conduct and interval | Concurrent vs sequential testing, data alignment |
| Statistical analysis guidance specificity | General algorithms, not prescriptive tests | General algorithms, not prescriptive tests | Property-specific expectations | Standards for reliability and error | Diagnostic metrics and flow considerations | Prescriptive tests for error, linearity, equivalence |

Supplementary Table 1. Comparison table between common risk of bias analysis tools.
